# Breast clinical target volume: HU-based glandular CTVs and ESTRO CTVs in modern and historical radiotherapy treatment planning

**DOI:** 10.1007/s00066-021-01839-5

**Published:** 2021-09-03

**Authors:** Marciana Nona Duma, Theresa Kulms, Stefan Knippen, Tobias Teichmann, Andrea Wittig

**Affiliations:** Department of Radiotherapy and Radiation Oncology; University Hospital, Friedrich Schiller University Jena, Bachstraße 18, 07743 Jena, Germany

**Keywords:** Breast cancer, Radiotherapy, adjuvant, Radiation coverage, Radiotherapy planning, computer-assisted, Breast-conserving surgery

## Abstract

**Purpose:**

The current study aimed to compare contouring of glandular tissue only (gCTV) with the clinical target volume (CTV) as defined according to European Society for Radiotherapy and Oncology (ESTRO) guidelines (eCTV) and historically treated volumes (marked by wire and determined by palpation and anatomic landmarks) in breast cancer radiotherapy.

**Methods:**

A total of 56 consecutive breast cancer patients underwent treatment planning based solely on anatomic landmarks/wire markings (“wire based”). From these treatment plans, the 50% and 95% isodoses were transferred as structures and compared to the following CT-based volumes: eCTV; a Hounsfield unit (HU)-based automatic contouring of the gCTV; and standardized planning target volumes (PTVs) generated with 1‑cm safety margins (resulting in the ePTVs and gPTVs, respectively).

**Results:**

The 95% isodose volume of the wire-based plan was larger than the eCTV by 352.39 ± 176.06 cm^3^ but smaller than the ePTV by 157.58 ± 189.32 cm^3^. The 95% isodose was larger than the gCTV by 921.20 ± 419.78 cm^3^ and larger than the gPTV by 190.91 ± 233.49 cm^3^. Patients with larger breasts had significantly less glandular tissue than those with small breasts. There was a trend toward a lower percentage of glandular tissue in older patients.

**Conclusion:**

Historical wire and anatomic landmarks-based treatment planning sufficiently covers the glandular tissue and the theoretical gPTV generated for the glandular tissue. Modern CT-based CTV and PTV definition according to ESTRO results in a larger treated volume than the historical wire-based techniques. HU-standardized glandular tissue contouring results in a significantly smaller CTV and might be an option for reducing the treatment volume and improving reproducibility of contouring between institutions.

## Introduction

Breast cancer is the most frequent cancer in women worldwide. Breast-conserving surgery should be followed by adjuvant radiotherapy (RT) [1]. In breast cancer RT, definition of the clinical target volume (CTV) is still the weakest part in development of a precise and personalized treatment [2, 3].

Treatment decisions taken nowadays in breast cancer are often based on trials from the 1980s, 1990s, and early 2000s. These randomized trials on adjuvant treatment were mostly based on two-dimensional (2D) RT [4, 5], which included clinical information, palpation of breast tissue, and marking with wire—without a standardized CTV [6]. The National Radiotherapy Trials Quality Assurance (RTTQA) group from the UK has carried out QA for breast RT trials, including START, FAST, SUPREMO, IMPORT LOW, IMPORT HIGH, and FAST FORWARD [5, 7]. The QA data for the START trials showed that most centers in the UK determined the breast dose distribution by planning on a 2D contour taken along the central plane of the breast [8]. In 1998, only two (<5%) START trial centers had access to a CT scanner for obtaining patient outlines, including information about the shape and position of the lung. Further eight had access to a CT simulation facility, which gave a limited number of slices (usually three) on which the dose distribution could be computed [5]. There was no standardized CTV contouring [9, 10]. Early studies on breast CTV demonstrated large uncertainties in contouring [11]. In 2005, when implementing CT-based contouring in their department, Struikmans et al. strongly recommended that each institute determine its interobserver variability with respect to the breast CTV before implementing the delineation of target volumes in daily practice [12]. In 2015, the European Society for Radiotherapy and Oncology (ESTRO) published a CT-based contouring guideline for breast CTV [2, 3]. However, many of the CT criteria presented therein are not strict. Furthermore, contouring of the breast CTV as recommended by ESTRO is not highly specific, because it also includes lobules of connective and fatty tissue; however, fatty tissue is not a risk region for breast cancer per se [13, 14]. Several other recent studies reported high variation in breast treatment planning with regard to the definition of target volumes, treatment planning margins, and the applied radiation technique [15, 16]. Thus, a more differentiated and standardized and less subjective contouring approach to breast tissue might be useful. Modern RT treatment planning systems allow for automated Hounsfield unit (HU)-based contouring. Thus, the glandular portion of the breast could be easily defined. Such an approach might have three important endpoints for breast cancer RT:

1. With clinical implementation of the more advanced dose calculation algorithms in modern RT planning, such as Monte Carlo, the specific tissue anatomy in terms of its elementary composition can be properly taken into account during treatment planning. The glandular proportion has a lower carbon but higher oxygen fraction than fat, and dose calculation becomes even more precise [17].
2. A standardized contouring approach can achieve better – more reproducible and less subjective – treatment planning in breast cancer.
3. Treating only the glandular tissue might result in even lower toxicities in breast cancer treatment.

The aim of this pilot study is to assess whether HU-based contouring is feasible and attainable. Furthermore, we assessed how volumes of wire/anatomic landmarks-based regions compare to the ESTRO CTV, as well as to contouring of the glandular proportion only.

## Materials and methods

All patients diagnosed with breast cancer between 2014 and 2018 were retrospectively screened for this study. Thereafter, 100 left-breast cancer patients were chosen arbitrary. Patients with breast implants or mastectomy were excluded, as were some patients for technical reasons. The remaining 56 patients were analyzed.

The field edge was marked with wire (by palpating the breast tissue) before the planning CT and treatment plans were calculated by experienced physicists without planning target volumes (PTVs), taking exclusively this information into account. All patients underwent “2D-analogous planning” based on wire markings/anatomic landmarks (such as the humeral head). Dose distributions were calculated using a collapsed-cone convolution superposition algorithm within the treatment planning system (Oncentra Masterplan; Elekta, Stockholm, Sweden). A typical plan consisted of up to six photon fields with mixed accelerating voltages (6 and 15 megavolts, MV) for the two main tangential irradiation angles. These tangent fields were shaped on the basis of wire markings/anatomic landmarks and were counterbalanced (medial vs. lateral and 6 MV vs. 15 MV) so that the buildup regions as well as the inner-lying tissues were covered with the desired homogeneous amount of dose. At least two of the four main resulting treatment fields contained virtual wedges to compensate for the curved anatomy of the irradiated breast. The 50 and 95% isodoses of these 2D-analogous plans were transferred as structures to the structure set and were defined as the reference in order to compare contoured volumes to the volumes that would have been treated without a contoured CTV.

Thereafter, all patients’ data were imported into the RayStation (V.8, RaySearch Laboratories, Stockholm, Sweden) planning software for evaluation. Herein, the left (tumor-affected breast after breast-conserving surgery) and right (healthy) breasts were contoured on the planning CT. The contouring was performed bilaterally in order to test the sensitivity of contouring by assessing the operated breast in comparison to the intact breast.

**Fig. 1:**
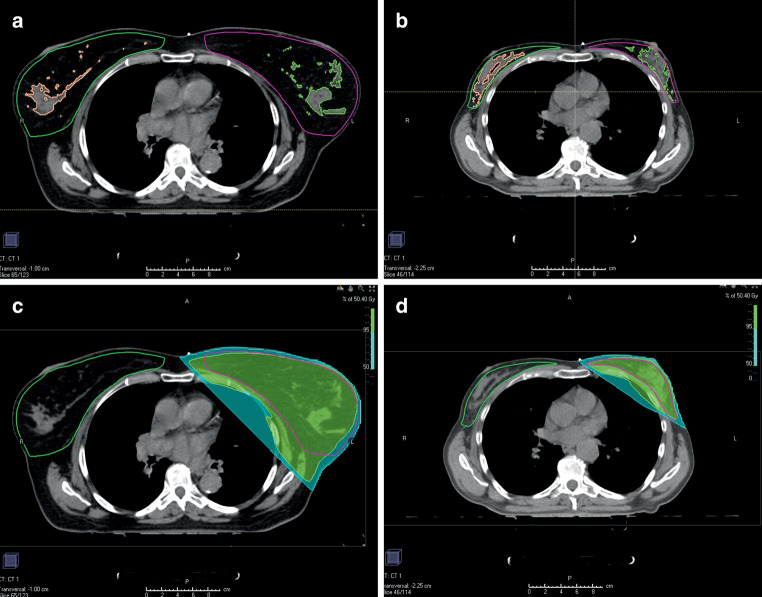
Exemplary contouring of the clinical target volume (CTV) defined by the European Society for Radiotherapy and Oncology guidelines (eCTV) and the glandular portion of the CTV (gCTV) for a patient with large breasts (**a**) and a patient with small breasts (**b**) and the corresponding wire defined 50% and 95% isodoses (**c** and **d**)

The contoured volumes were (Fig. 1):

1. A CTV contoured according to the ESTRO guideline (eCTV) [2, 3] on both sides: on the left, affected ipsilateral side (eCTVipsi) as well as on the contralateral side (eCTVcontra).
2. The volume of the glandular portion (gCTV) of the ipsilateral (gCTVipsi) and contralateral breast (gCTVcontra). For this, automatic contouring of the glandular tissue was performed in the RayStation software, discriminating the fatty tissue from the glandular tissue by the HU values of −59 (fat tissue was defined by HU < −59, glandular tissue by HU ≥ −59) [13].

Standardized CTV-to-PTV safety margins (+1 cm, adapted within the external contour and by subtracting the lungs) were applied to the eCTV and the gCTV to result in ePTV and gPTV, respectively.

Patients were stratified by age (≤ 60 years and > 60 years) [18–24] and by breast volume/bra size [25]. As only volumetric data on CTV/PTV volumes were available, it was decided to stratify patients according to the available literature. Very few studies have investigated the relationship between breast volume and bra size [25–27]. Table 1 presents the volume in millimeters and the corresponding cup sizes. For the current study it was decided to classify breasts into smaller or larger breasts by the threshold of cup D/399 ml.

**Table 1:** Breast volume and cup sizes (adapted from [17])

Breast volume	Cup size
150–249 ml	70B/A
250–299 ml	70B/C, 75 B/C
300–349 ml	70C, 75 C/D, 80 C
350–399 ml	70D, 75D
400–499 ml	70E, 75D, 75DD, 80C/D
500–599 ml	70E, 75DD, 80D
600–699 ml	70DD/E, 75DD/E, 80D
700–799 ml	75E/F, 80DD, 85D
800–999 ml	75F, 80E/F, 85D
1000–1099 ml	70G, 75F, 80E/F, 90D
1100–1499 ml	75G, 85E, 90DD
1500–2000 ml	80H, 85G, 90E

Taking the laterality into account, gCTV, eCTV, gPTV, and ePTV were analyzed. Moreover, it was analyzed whether wire-based-treatment planning would treat the same or a larger region as compared to CT-based contouring as recommended by ESTRO. In order to assess coverage of the CTVs and PTVs by the wire-based-planning dose distributions, the 50 and 95% isodoses were converted into structures. These structures were volumetrically compared to the CTVs and PTVs.

As a measure of congruence between the shape of the target volumes and the reference isodose volumes, the Dice conformity index was assessed [28].

$$DSC=\frac{2\mathrm{xTV}_{RI}}{TV+V_{RI}}$$

TV_RI_: target volume covered by the reference isodose
TV: volume of target volume
V_RI_: volume of the reference isodose

The Dice conformity index is based on the distance between the surface of the treatment volumes and the volume of the reference isodose. This allows evaluation of the congruence in shape between target volumes and the volume of the reference isodose, but additionally of the degree of target coverage and sparing of normal tissue [28].

The statistical analyses were performed with IBM SPSS Statistics 26 (IMB Corp., Armonk, NY, USA). Reported are mean ± standard deviation and median (with range) values. Correlations between the eCTV and wire-based planning as well as between the ePTV and gPTV were analyzed by *t-*test. A value of *p* < 0.05 was considered to be statistically significant.

## Results

There was no statistical difference between the ipsilateral eCTV and the contralateral eCTV. The operated breasts appeared not to be significantly smaller than the healthy breasts (difference of 18.33 ± 111.87 cm^3^; *p* = 0.225). Table 2 depicts the volumes of eCTVs and gCTVs.

**Table 2:** Absolute volumes of clinical target volumes (CTVs) according to the ESTRO guideline (eCTV) and glandular portion CTVs (gCTV) for both sides

	eCTVipsi (cm^3^)	eCTVcontra (cm^3^)	gCTVipsi (cm^3^)	gCTVcontra (cm^3^)
Median	632.22	617.76	75.05	59.53
Minimum	109.37	111.75	27.67	15.49
Maximum	1707.74	1481.21	315.86	295.50

*eCTVipsi* ESTRO CTV on the ipsilateral site,* eCTVcontra* ESTRO CTV on the contralateral site,* gCTVispi* glandular tissue CTV,* gCTVcontra* glandular tissue CTV on the contralateral site

Table 3 depicts the volumes of the PTVs generated from both CTVs.

**Table 3:** Volumes of planning target volumes (PTVs) generated based on the ESTRO clinical target volume (eCTV) and the glandular tissue CTV (gCTV) with a margin of 1 cm

	ePTVipsi (cm^3^)	ePTVcontra (cm^3^)	gPTVipsi (cm^3^)	gPTVcontra (cm^3^)
Median	1137.00	1131.94	762.12	723.75
Minimum	369.32	373.63	296.69	288.18
Maximum	2499.62	2272.69	1713.00	1627.97

*ePTVipsi* ESTRO PTV on the ipsilateral site,* ePTVcontra* ESTRO PTV on the contralateral site,* gPTVispi* glandular tissue PTV,* gPTVcontra* glandular tissue PTV on the contralateral site

Of the 56 patients, 15 had a smaller (≤ 399 cm^3^) and 41 a larger breast size (> 399 cm^3^).

**Fig. 2:**
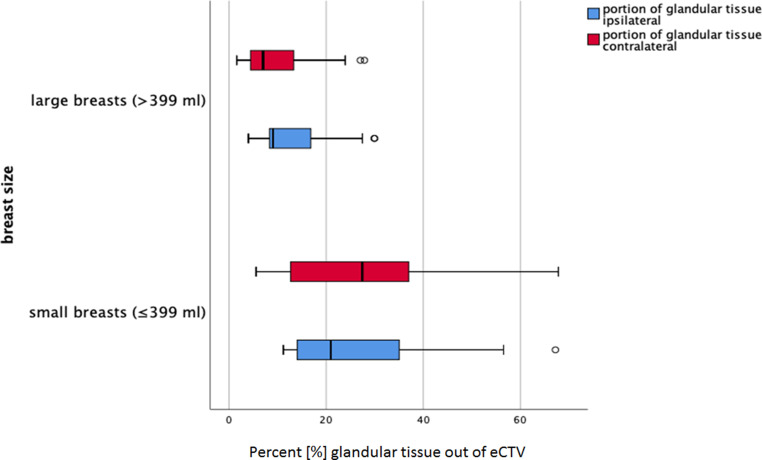
Glandular proportion within the clinical target volume (CTV) contoured according to ESTRO guidelines (*eCTV*)

The relative glandular proportion of the breast was significantly larger in patients with small breasts as compared to patients with large breasts. In the patient group with small breasts, the eCTV contained up to 27.7% glandular tissue on the ipsilateral site and 29% on the contralateral site. In the patient group with large breasts, the glandular tissue comprised up to 12.8% of the ipsilateral and 9.9% of the contralateral eCTV (*p* = 0.003; Fig. 2).

Of the total study cohort, 30 patients were older than 60 and 26 patients were ≤ 60 years. Comparing the two age groups—younger (≤ 60 years) and older (> 60 years) patients—there was a trend toward a higher percentage of glandular tissue within the eCTV in younger patients. The mean glandular portion was 20.32 ± 16.04% of the eCTVipsi for younger patients and 13.71 ± 7.47% of eCTVipsi for older patients. Similarly, the mean glandular portion of the contralateral breast was 19.26 ± 19.13% of the eCTVcontra for younger patients and 11.29 ± 8.40% of eCTVcontra for older patients (*p* = 0.06 ipsilateral,* p* = 0.058 contralateral).

The 50% isodose was significantly both larger than the eCTV (by a mean of 891.06 ± 286.72 cm^3^) and larger than the ePTV (by 381.09 ± 242.01 cm^3^), respectively. The 95% isodose was larger than the eCTV by 352.39 ± 176.06 cm^3^, but smaller than the ePTV by 157.58 ± 189.32 cm^3^.

Regarding the glandular tissue, the 50% isodose was significantly larger than the gCTV (by 1459.87 ± 555.21 cm^3^) and the gPTV (by 729.58 ± 339.71 cm^3^). The 95% isodose was larger than the gCTV (by 921.20 ± 419.78 cm^3^) and larger than the gPTV by (190.91 ± 233.49 cm^3^).

**Fig. 3:**
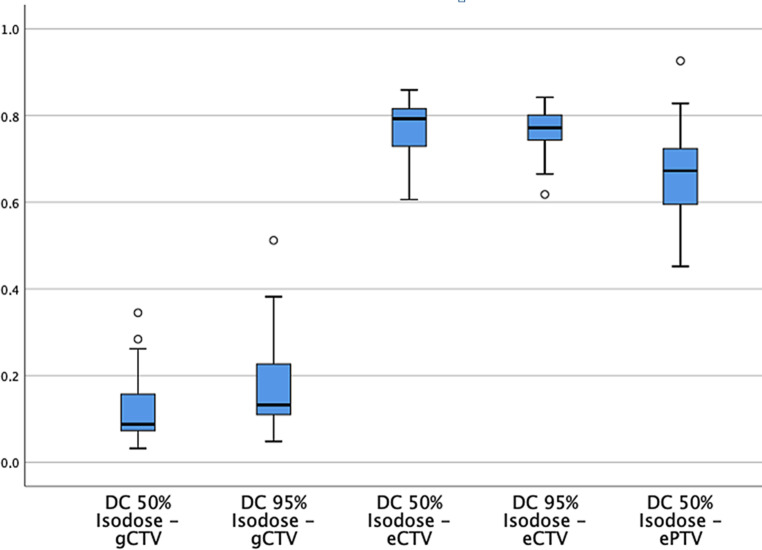
Dice conformity index (DC)

The Dice conformity index showed a worse similarity to the gCTV than to the eCTV or ePTV (Fig. 3).

## Discussion

The current study was able to demonstrate that wire-based treatment planning sufficiently covered the ESTRO CTV (eCTV), the glandular tissue (gCTV), and the theoretical PTV generated for the glandular tissue (i.e., the gPTV in this study). However, compared to a volume that would nowadays be treated (i.e., ePTV = eCTV + 1 cm), the wire-based planning based on anatomic landmarks and wire marking resulted in a smaller treated volume (by approximately 160 cm^3^).

Bentel et al. [11] analyzed the optimal, CT-based tangential field as compared to wire markers in 108 patients. They concluded that the glandular tissue (not specifically delineated in the study of Bentel et al.) is visualized farther laterally/posteriorly and deeper on the CT scan than it was appreciated clinically. This is similar to the current data and shows that clinically assessing the treatment fields results in smaller treated volumes. Nonetheless, some questions could not be answered by Bentel et al. For e.g., was the glandular tissue really not treated just because the treament fields seem smaller? The current study was able to demonstrate that even if the whole breast PTV is larger when CT contouring is perfomed, the glandular tissue would have still been treated (i.e., the gPTV in our study) in the wire-based era. The size difference between gPTV and ePTV is significant, but maybe clinically we could argue that it is not relevant. Nonetheless, the gCTVispi is 10 times smaller that gPTVispi and the eCTV is almost as large as the gPTV. This is a clinically highly relevant issue. Modern RT could treat smaller volumes (i.e., with smaller safety margins) and should be considered. However, this might be an issue in breast cancer patients, as, e.g., image-guided radiotherapy (IGRT) is not primarily considered a necessary tool in breast cancer. For example, IGRT was still an issue when the IMPORT HIGH trial opened, as only four centers were able to deliver the required RT for breast patients [5].

Another important question that arises from this study and that by Bentel et al. [11] is whether modern RT can and should treat a different volume than the ESTRO CTV. A more in-depth analysis is necessary to theoretically answer this question.

The present study showed significant differences, with more glandular tissue in smaller breasts than in larger breasts and a trend toward a higher percentage of glandular tissue in younger patients than in older patients. A study performed at the Mayo Clinic on a cohort of 13,455 women has shown that progression of lobular involution over time is associated with a decreased breast cancer risk [23]. Might this also be relevant for treatment of breast cancer? It is known that older patients profit less from adjuvant radiation to the whole breast than younger patients [4]. No clinical data are yet available on the impact of lobular involution on outcomes after treatment of older patients, but this would be an issue worth assessing. On the other hand, we could show in this pilot study that larger breasts have a lower proportion of glandular tissue—approximately 10–13% of the total eCTV. Larger breasts are mostly found in older patients, and it is known that a large breast is a risk factor for higher acute toxicities during RT [29].

Combining the information on the glandular portion—paired with lobular involution in older patients and the knowledge that in wire-based radiotherapy, clinically defined volumes are smaller than in 3D-CRT—might open a new approach to treatment of older patients with large breasts. Modern RT techniques allow high conformity within the planning target volume [30]. Thus, in principle, a gPTV might be feasible for these patients, with an approximately 10-fold reduction of the CTV (from 600 cm^3^ eCTV to 75 cm^3^ gCTV).

A significant reduction of the PTV is of course also achieved by partial breast irradiation (PBI) as shown, e.g., by the IMPORT LOW study [31]. In this study, the whole breast volume, defined by scrolling through CT slices, was used to derive a field-based approach for PBI. Nonetheless, delineation of breast tissue—as stated by the authors of the IMPORT LOW trial—is very difficult and could result in overestimation of the whole breast volume. An HU-based approach might help to standardize and better define the regions for PBI performed according to the IMPORT LOW.

Limitations of the current pilot study are the low number of patients and its retrospective and monocentric nature. For example, it is impossible to assess whether other radiation oncologists would have wire marked the breast similarly. The HU-based contouring in this study was performed according to the data of Fogliata et al. [13]. Automatic contouring has not yet been validated in clinical trials and must be considered as work in progress [32].

## Conclusion

Historical wire and anatomic landmarks-based treatment planning sufficiently covers the glandular tissue as well as the theoretical gPTV generated for the glandular tissue. Modern CT-based CTV and PTV definition according to ESTRO results in a larger treated volume than the historical wire-based techniques. HU-standardized glandular tissue contouring results in a significantly smaller CTV and might be an option for reducing the treatment volume and improving reproducibility of contouring between institutions.
